# Genome Assembly and Comparative Analysis of the Egg Parasitoid Wasp *Trichogramma dendrolimi* Shed Light on the Composition and Evolution of Olfactory Receptors and Venoms

**DOI:** 10.3390/insects14020144

**Published:** 2023-01-31

**Authors:** Xue Zhang, Zhuo Jiang, Xilin Jiao, Yang Yu, Zhenan Wang, Yangyang Hou, Guohua Duan, Wenmei Du, Changchun Ruan, Junjie Zhang, Ying Hu

**Affiliations:** 1 Engineering Research Center of Natural Enemies, Institute of Biological Control, Jilin Agricultural University, Changchun 130118, China; 2 Department of Plant Pathology, College of Plant Protection, Jilin Agricultural University, Changchun 130118, China; 3 Department of Entomology, College of Plant Protection, Jilin Agricultural University, Changchun 130118, China

**Keywords:** egg parasitoid wasp, *Trichogramma dendrolimi*, genome assembly, olfactory receptor, venom

## Abstract

**Simple Summary:**

*Trichogramma* wasps are minute egg parasitoids that are one of the most used biocontrol agents to manage lepidopteran pests. Given the great success in industrializing a mass-rearing method for the cost-effective production of *Trichogramma dendrolimi*, this parasitoid wasp has been used extensively to control agricultural and forestry pests in China. So far, while the biology and ecology of *T. dendrolimi* have been intensively studied to provide successful biological control, the limited genome information constrains the interpretation of the molecular mechanism underlying its host recognition and parasitism. Here, we assembled a high-quality reference genome for *T. dendrolimi* and identified the repetitive sequences and protein-coding genes. A phylogenomic tree constructed for *T. dendrolimi* and 24 hymenopteran species places the family Trichogrammatidae sister in the remaining Chalcidoidea species. We found that significantly expanded and contracted gene families are involved in the development, regulation and transport processes in *T. dendrolimi*, respectively. Additionally, 24 odorant binding proteins, 100 olfactory receptors, 27 gustatory receptors, 27 ionotropic receptors and 87 venom genes were identified in the genome of *T. dendrolimi*. Our study provides a foundation for future studies on the molecular mechanism underlying host recognition and parasitism in the *Trichogramma* species.

**Abstract:**

*Trichogramma dendrolimi* is one of the most successfully industrialized *Trichogramma* species used to control agricultural and forestry pests in China. However, the molecular mechanisms underlying its host recognition and parasitism remain largely unknown, partially due to the limited genome information of this parasitoid wasp. Here, we present a high-quality de novo assembly of *T. dendrolimi* through a combination of Illumina and PacBio sequencing technologies. The final assembly had a length of 215.2 Mb and contains 316 scaffolds with a scaffold N50 size of 1.41 Mb. Repetitive sequences with a length of 63.4 Mb and 12,785 protein-coding genes were identified. Significantly expanded gene families were identified to be involved in the development and regulatory processes, while remarkably contracted gene families were involved in the transport processes in *T. dendrolimi*. The olfactory and venom-associated genes were identified in *T. dendrolimi* and 24 other hymenopteran species, using uniform methods combining BLAST and HMM profiling. The identified venom genes of *T. dendrolimi* were enriched in antioxidant activity, tricarboxylic acid cycle, response to oxidative stress and cell redox homeostasis. Our study provides an important resource for comparative genomics and functional studies to interpret the molecular mechanisms underlying host recognition and parasitism of *Trichogramma* species.

## 1. Introduction

*Trichogramma* (Hymenoptera: Trichogrammatidae) wasps are minute egg parasitoids (<1 mm in length) that are one of the most used biocontrol agents to control lepidopteran pests [1,2,3]. In China, after decades of efforts on improving the technology of mass rearing and the inundative release of the *Trichogramma* species, these egg parasitoid wasps have been widely utilized to control Asian corn border (ACB) *Ostrinia furnacalis* (Lepidoptera: Crambidae) [4], rice stem borer *Chilo suppressalis* (Lepidoptera: Crambidae) [5], oriental fruit moth *Grapholita molesta* (Lepidoptera: Tortricidae) [6], oriental armyworm *Mythimna separata* (Lepidoptera: Noctuidae) [7] and moth *Heortia vitessoides* (Lepidoptera: Pyralidae) [8]. *Trichogramma dendrolimi* is one of the most successfully industrialized *Trichogramma* species to control agricultural and forestry pests because of its broad distribution in China, the wide host range and optimized mass-rearing method on eggs of the Chinese oak silkworm, *Antheraea pernyi* (Lepidoptera: Saturniidae) [4,9]. In northeast China, *T. dendrolimi* mass-reared on eggs of *A. pernyi* have been successfully applied to control ACB through wide-scale inundative releases, resulting in 70% of ACB eggs being parasitized in the first generation, which has become one of the widely used biocontrol agents in integrated pest management programs [9]. In the major corn-growing regions, the area of corn protected from ACB with inundative releases of *T. dendrolimi* has increased nine times over the past decade, accounting for 35% of the corn production area in northeast China [9].

The biocontrol research of the *Trichogramma* species has been fruitful on many applied aspects, such as host location, host suitability, parasitic ability and breeding behavior to improve its biological control and mass-rearing efficiency [9]. For instance, the ultrastructure of the sensilla of female and male *T. dendrolimi* showed that there were different types of sensilla on the antenna, eyes, mouthparts, wings, legs and external genitalia, which provided a fundamental understanding of the sensory mechanisms in *T. dendrolimi* [10]. Recent transcriptomic analyses of the *Trichogramma* species identified several olfactory receptors (ORs), gustatory receptors (GRs), ionotropic receptors (IRs) and odorant binding proteins (OBP)-related genes, providing genetic bases to further study the olfactory systems of the *Trichogramma* species [11,12,13]. In addition, the suitability of agricultural pests and factitious hosts has been evaluated to increase the effectiveness of biological control and develop more efficient mass-rearing techniques in recent years [7,14]. The host egg age is one of the key factors affecting the parasitism of the *Trichogramma* species. Many studies have shown that *Trichogramma* females usually showed high fitness on young host eggs, which may provide more available essential nutrients to support the development of the parasitoid offspring or have less competitive host embryos [7,15]. However, such a phenomenon has not been observed when *Trichogramma* females parasitize their highly suitable hosts [16]. Besides *T. dendrolimi*, *T. ostriniae* has also been widely used as a biological agent to control ACB in most of corn-growing regions in China. However, the selection of the best *Trichogramma* species to control ACB in the field remains a practical challenge that cannot be well addressed with the current knowledge. Compared with *T. ostriniae*, *T. dendrolimi* is a less effective biocontrol agent because it prefers to parasitize young ACB eggs (0–8 h old), while *T. ostriniae* can parasitize 0–48 h-old ACB eggs [16]. Interestingly, the parasitism ability of *T. dendrolimi* on ACB eggs pre-parasitized by irradiated *T. ostriniae* females could be significantly enhanced, indicating that it is likely that the host-specific venom from *T. ostriniae* is more effective to counteract the immune response of ACB eggs [17]. So far, while the mass rearing and release of *Trichogramma* species for decades has achieved great success in providing an environmentally friendly and cost-effective approach to reduce the abundance and damage of lepidopterous pests in the field, the molecular mechanisms underlying the host location and parasitism in the *Trichogramma* species remain largely unknown, partially due to limited genome information of this parasitoid wasp.

Whole-genome information could advance a deep understanding of the genetic and evolutionary bases for physiological biology and parasitism strategies of parasitoid wasps, facilitate mapping and cloning the key quantitative traits loci and provide insights to further increase the efficiency and effectiveness of biocontrol by utilizing parasitoid wasps as pest insect control agents. Since the first three *Nasonia* species’ genomes initiated a new era of genomic studies of parasitoid wasps [18], an increasing number of parasitoid wasps have been whole-genome sequenced, due to their unique haplodiploid mating system and development of revolutionary sequencing technologies [19]. To the best of our knowledge, two draft *Trichogramma* genomes have been published to date, including *T. pretiosum* and *T. brassicae* [20,21]. The genomic study on *T. pretiosum* showed that its specialized egg parasitoid lifestyle and adaptation to miniaturization may be due to the rapid evolution of its genome [20]. Comparative analysis of *T. pretiosum* and *T. brassicae* revealed that around 10% of ortholog clusters were specific to each of the two *Trichogramma* genomes [21]. Another *Trichogramma* species (*Trichogramma evanescens*, GenBank assembly accession: GCA_902732785.1) available on NCBI is highly fragmented. Recent studies have shown that parasitoid wasps exhibit exceptional levels of variations in gene content and gene regulation among different species [22,23]. Even among closely related parasitoid species they showed large genetic variations, which may contribute to the variable mechanisms of parasitism [24,25,26]. This limits the utility of the *T. pretiosum* genome or the *T. brassicae* genome for interpreting data derived for species-specific features in a *T. dendrolimi* genetic background. The whole genome sequence of the *T. dendrolimi* is not only critical to fully exploit the various biological process in *T. dendrolimi*, but also enriches the genetic resources of *Trichogramma* species to perform the comparative genomic analysis.

In this study, we present a high-quality reference genome of *T. dendrolimi* through a combination of Illumina short-read sequencing and PacBio long-read sequencing technologies. Phylogenetic analysis revealed the genetic relationships among the *Trichogramma* species, 18 representative Chalcidoidea species and 3 outgroup hymenopteran species with publicly available genomes. Significantly expanded and contracted gene families in the *T. dendrolimi* genome were investigated. The olfactory and venom-associated genes were identified in *T. dendrolimi* and 24 other hymenopteran species with uniform methods combining BLAST and HMM profiling. Our study provides an important resource for comparative genomics and functional studies to interpret the molecular mechanism underlying host recognition and parasitism in *Trichogramma* species.

## 2. Materials and Methods

### 2.1. Animals

*T. dendrolimi* were collected from the corn fields in Yitong, Jilin Province, China (125°11′ E, 43°3′ N) in 2015 and were confirmed through the morphological characteristics of male genital capsules. The wasp population was reared on the eggs of *Antheraea pernyi* (fresh eggs were dissected from the ovaries of female *A. pernyi* and supplied in glass tubes to newly emerged *T. dendrolimi* for oviposition). The eggs were kept at 26 °C ± 1 °C, 70% ± 5% relative humidity (RH) with a 16:8 h (L:D) photoperiod.

### 2.2. PacBio and Illumina Data Generation

The line used for sequencing was created from an individual mother and kept in the laboratory for more than three years without outcrossing with other lines. Given the tiny body size of *T. dendrolimi* and the need for a large amount of DNA for sequencing, genomic DNA was extracted from more than 10,000 haploid male pupae of *T. dendrolimi* dissected from the eggs of *A. pernyi* using a DNeasy Blood and Tissue Kit (Qiagen). We utilized the Illumina HiSeq 2000 (Illumina HiSeq2000, RRID:SCR_020132) and PacBio platforms (PacBio Sequel II System, RRID:SCR_017990) to sequence the genome of *T. dendrolimi.* Large insert (20 Kb) SMRTbell libraries were prepared and sequenced using the PacBio Sequel system according to the recommended protocol, yielding a total of 16.86-Gb high-quality long reads. DNA extracted from the same sample was used to build Illumina sequencing libraries. Five paired-end libraries with average insert sizes of 250 bp, 450 bp, 2 kb, 5 kb and 10 kb were constructed for genomic survey and sequenced with pair-end 150 bp on the Illumina HiSeq 2000 platform to yield 44.04 Gb (~200-fold coverage) short reads for error correction.

### 2.3. Genome Assembly

The PacBio SMRT subreads were error corrected and assembled using Canu v1.8 (Canu, RRID:SCR_015880) using default parameters [27]. To improve the accuracy of the reference assembly, Arrow v2.3.3 [28] was used to correct the sequencing errors with default parameters. The Illumina pair-end reads were mapped to the corrected contigs above with BWA mem [29] with default parameters; high-quality mapped reads were further used to polish the assembly with Pilon v1.2.3 (Pilon: RRID:SCR_014731) [30] with the default parameters.

### 2.4. Assessment of the Genome Completeness and Quality

The genome completeness and quality were assessed using the benchmarking universal single-copy orthologs (BUSCO) v3.02 (BUSCO: RRID:SCR_015008) [31]. The assembly was tested against the Insect BUSCO “insecta_odb10” database, which contained 1367 bench-marking universal single-copy orthologous genes.

### 2.5. Genome Annotation

Repetitive sequences of the *T. dendrolimi* genome were identified using a combination of de novo and homology searches for known repeat databases. Briefly, the homology searches were carried out by screening the RepeatMasker v4.0.5 (RepeatMasker: RRID:SCR_012954) [32] against the public arthropod set of Repbase (Repbase: RRID:SCR_021169) [33] and the transposable element protein database with RepeatProteinMask v4.0.5 (www.repeatmasker.org, accessed on 12 December 2021). For de novo predication, the repeat library was constructed using RepeatModeler v1.0.7 [34] (RepeatModeler: RRID:SCR_015027) with default parameters. Then, RepeatMasker was employed to align sequences from the *T. dendrolimi* genome assembly to the de novo library for identifying repetitive sequences. Tandem repeats were also searched using Tandem Repeats Finder v4.09 (Tandem Repeats Finder: RRID:SCR_022193) [35]. All repeat sequences were masked before genome assembly.

A combination of protein-homology-based predication, ab initio prediction and transcriptome-based methods were utilized to predict the protein-coding genes in the *T. dendrolimi* genome. For homology-based predication, protein sequences from five sequenced insect species, including *Nasonia vitripennis*, *Apis mellifera*, *Apis cerana*, *Trichogramma pretiosum* and *Drosophilia melanogaster*, were aligned against the *T. dendrolimi* genome using TBLASTN v2.8.1 (NCBI BLAST, RRID:SCR_004870) [36] (E-value < 1 × 10^−5^) and the genome sequences were aligned against the matching protein sequences using Genewise v2.4.1 (Genewise: RRID:SCR_015054) [37] to define the gene models. For ab initio gene prediction, AUGUSTUS v3.3.2 (AUGUSTUS: RRID:SCR_008417) [38] and SNAP v2006-07-28 (SNAP: RRID:SCR_007936) [39] were performed on the repeat-masked genome with parameters trained on 1000 randomly selected genes from *T. pretiosum*. For the transcriptome-based method, our previous published RNA-seq reads [12] were aligned to the assembly of *T. dendrolimi* by Tophat2 (Tophat: RRID:SCR_013035) [40] to identify candidate exon regions and the donor and acceptor sites. The alignments were assembled into transcripts using Cufflinks v2.2.1 (Cufflinks: RRID:SCR_014597) [41]. The de novo transcriptome assembly was performed using Trinity v2.8.4 (Trinity: RRID:SCR_013048) [42] with default parameters. The resulting Cufflinks and Trinity assemblies were applied to PASA v2.2.0 (PASA: RRID:SCR_014656) [43] to build a comprehensive transcriptome database. Finally, all the predication results were combined by EvidenceModeler (EVM) v1.1.1.(EvidenceModeler: RRID:SCR_014659) [44] to build a consensus gene set. All predicted genes were annotated based on homologue searches and the best match to the databases of KEGG (KEGG: RRID:SCR_012773) [45], InterPro (InterPro: RRID:SCR_006695) [46], Swiss-Prot (UniProtKB/Swiss-Prot: RRID:SCR_021164) [47] and NCBI-NR using BLASTP (BLASTP: RRID:SCR_001010) (e-value < 1 × 10^−5^). The annotation information from different sources was combined for each gene in the final gene annotation set.

Noncoding RNAs, including transfer RNA (tRNA), microRNA(miRNA), ribosome RNA (rRNA) and small nuclear RNA (snRNA), were identified using the following method: rRNA, miRNA and snRNA were annotated by mapping against the Rfam database (Rfam: RRID:SCR_007891) [48] using BLASTN (BLASTN: RRID:SCR_001598); tRNA was predicted using tRNAscan-SE v2.0 (tRNAscan-SE: RRID:SCR_010835) [49]. In addition, rRNA and subunits were predicted using RNAMMER v1.2 (RNAMMER: RRID:SCR_017075) [50].

### 2.6. Phylogenetic Analysis

The protein sequences of 25 hymenoptera genomes were downloaded from InsectBase 2.0 [51] and NCBI, including *Apis mellifera* (Amel) (GenBank assembly accession: GCA_003254395.2), *Orussus abietinus* (Oabi) (GenBank assembly accession: GCA_000612105.2), *Athalia rosae* (Aros) (GenBank assembly accession: GCA_000344095.2), *Copidosoma floridanum* (Cflo) (GenBank assembly accession: GCA_000648655.2), *Trichogramma pretiosum* (Tpre) (GenBank assembly accession: GCA_000599845.3), *Trichogramma brassicae* (Tbra) (GenBank assembly accession: GCA_902806795.1), *Trichogramma evanescens* (Teva) (GenBank assembly accession: GCA_902732785.1), *Ormyrus nitidulus* (Onit) (GenBank assembly accession: GCA_900474335.1), *Ormyrus pomaceus* (Opom) (GenBank assembly accession: GCA_900474385.1), *Eurytoma adleriae* (Eadl) (GenBank assembly accession: GCA_900480045.1), *Eurytoma brunniventris* (Ebru) (GenBank assembly accession: GCA_900475205.1), *Torymus auratus* (Taur) (GenBank assembly accession: GCA_900474315.1), *Torymus geranii* (Tger) (GenBank assembly accession: GCA_900474355.1), *Megastigmus dorsalis* (Mdor) (GenBank assembly accession: GCA_900490025.1), *Megastigmus stigmatizans* (Msti) (GenBank assembly accession: GCA_900490015.1), *Eupelmus urozonus* (Euro) (GenBank assembly accession: GCA_900480035.1), *Eupelmus annulatus* (Eann) (GenBank assembly accession: GCA_900480025.1), *Cecidostiba fungosa* (Cfun) (GenBank assembly accession: GCA_900474305.1), *Cecidostiba semifascia* (Csem) (GenBank assembly accession: GCA_900474235.1), *Pteromalus puparum* (Ppup) (GenBank assembly accession: GCA_012977825.2), *Trichomalopsis sarcophagae* (Tsar) (GenBank assembly accession: GCA_002249905.1), *Nasonia vitripennis* (Nvit) (GenBank assembly accession: GCA_009193385.2), *Nasonia giraulti* (Ngir) (GenBank assembly accession: GCA_000004775.1) and *Nasonia longicornis* (Nlon) (GenBank assembly accession: GCA_000004795.1). OrthoFinder v2.5.1(OrthoFinder: RRID:SCR_017118) [52] was used to identify the orthologous and paralogous genes families. The protein sequences of the identified single-copy genes were aligned by MAFFT v7.453 (MAFFT: RRID:SCR_011811) [53]. The best-fit amino acid substitution model—JTT + G + F—was determined using ProTest v.3.4.2 under AIC criteria [54]. RAxML v8.2.9 (RAxML: RRID:SCR_006086) with the maximum likelihood method was used to construct a phylogeny [55] using 1000 replicates to calculate bootstrap support. The MCMCTree program in the software package PAML v.4.9 (PAML: RRID:SCR_014932) was used to estimate the divergence time [56], using divergence time calibrated from the TIMETREE database (TIMETREE: RRID:SCR_021162) [57]. The minimum and maximum divergence times between *A. mellifera* and *O. abietinus* were 235–240 million years ago (Mya) [57].

### 2.7. Gene Family Expansion and Contraction Analysis

CAFÉ (Computational Analysis of Family Evolution) v4.2.1 (CAFÉ: RRID:SCR_005983) was used to analyze the gene family expansion and contraction [58,59]. The results from the gene family clustering from OrthoFinder and a phylogenetic tree with divergence time were used as inputs to investigate the ancestral gene content of each cluster at each node. GO enrichment analysis was conducted for gene families with significant expansion and contraction in the *T. dendrolimi* genome with Viterbi *p*-values < 0.05, using the Bioconductor package topGO (version 2.32.0) (topGO: RRID:SCR_014798) [60]. In the topGO analysis, GO terms significance of interest were assessed based on Fisher’s exact test statistic, using 0.05 as the significance threshold.

### 2.8. Gene Family Annotation

The gene families of olfactory receptors (ORs), odorant binding proteins (OBPs), gustatory receptors (GRs), ionotropic receptors (IRs) and venom proteins were manually annotated. The known orthologous protein sequences of *Apis mellifera* and several parasitoid wasps, including *Pteromalus puparum*, *Nasonia vitripennis*, *Chelonus inanitus*, *Leptopilina boulardi* and *Leptopilina heterotoma* from NCBI GeneBank and Hidden Markov models (HMMs), were used as references for gene identification. BLASTP v.2.7.1 (e-value < 1 × 10^−5^, bit score > 100, identity > 70% and query coverage > 70%) (BLASTP: RRID:SCR_001010) and HMMER v3.3.2 (HMMER: RRID:SCR_005305) [61] were used to search the candidate genes in the genomes of *T. dendrolimi* and 24 other hymenopteran species. The annotated candidate gene sequences were aligned using MAFFT v7.453 [53]. IQ-TREE v1.6.11 (IQ-TREE: RRID:SCR_017254) [62] was used to construct the phylogenetic tree (-m TEST -bb 1000 -alrt 1000).

## 3. Results

### 3.1. Genome Sequencing and Assembly

We assembled a high-quality genome of *T. dendrolimi* through single-molecule real-time (SMRT) sequencing with the PacBio Sequel platform and paired-end sequencing with the Illumina HiSeq platform. The assembly of 16.86 Gb PacBio long reads, representing ~75-fold sequencing coverage of the *T. dendrolimi* genome, resulted in a 215.2 Mb assembly containing 316 scaffolds with a scaffold N50 size of 1.41 Mb (Table 1 and Table S1). Approximately 95.15% of 44.02 Gb Illumina paired-end reads generated with libraries constructed from five different insert sizes (Table S2) was aligned and covered 98.18% of the assembled genome, suggesting that the *T. dendrolimi* genome was sufficiently covered by the assembly. The above Illumina paired-end reads were also used to polish the assembled contigs. The quality and completeness of the *T. dendrolimi* genome was evaluated through BUSCO analysis, which showed that 93.4% (1276), 1.0% (13) and 5.6% (78) of the 1367 BUSCO genes within the insecta_odb10 BUSCO set were present in the assembled *T. dendrolimi* genome as complete, fragmented and missing genes, respectively (Table 1). The total assembly size of the *T. dendrolimi* genome was very similar to the genome of *T. evanescens* but approximately 20 Mb bigger than that of *T. pretiosum* and 20 Mb smaller than that of *T. brassicae*. The genome size of the four *Trichogramma* species were moderate in Chalcidoidea compared with those of other genomes available on InsectBase 2.0 [51] (Figure S1). The GC content of the *T. dendrolimi* genome and three other *Trichogramma* species genomes were very similar (39.8–39.9%) (Table S1). Such low GC content is a common feature of Chalcidoidea genomes (Figure S1).

### 3.2. Genome Annotation

Using all of the protein-homology-based predication, ab initio prediction and transcriptome-based methods, we predicted 12,785 high-confidence protein-coding genes, with an average gene length, coding sequence length, average exon length, average intron length and exon number of 6407.61 bp, 1389.05 bp, 268.47 bp, 1202.33 bp and 5.17, respectively. The values of all parameters of *T. dendrolimi* gene models except average intron length were smaller than those of *T. pretiosum* (Table S3). In addition, 11,657 (91.2%) were functionally annotated using public databases (Table S4).

Our pipeline identified that 29.46% of the *T. dendrolimi* genome was covered by the repetitive sequences with a length of 63.4 Mb (Table S5). The most prevalent super family was retrotransposon (17.63%), counting for 37.9 Mb of the genome. Among the retrotransposon, long terminal repeat (LTR) Gypsy-like retrotransposons and long interspersed nuclear elements (LINEs) were the two prevalent super families, which were predicted to cover 10.97% and 6.55% of the genome, respectively. In addition, DNA transposon was predicated to occupy 6.41% of the genome, accounting for 13.8 Mb of the genome. There were some unclassified (here denoted as others) or unknown repetitive sequences in the *T. dendrolimi* genome, which covered 0.00036% and 8.11% of the genome. The fraction of the repetitive sequences was very similar to that of *T. pretiosum* (30.3%), which was predicted using the frequency of k-mers [20].

Non-coding RNAs were identified in the genome of *T. dendrolimi*, including transfer RNA (tRNA), microRNA (miRNA), ribosome RNA (rRNA) and small nuclear RNA (snRNA). There were 1140 non-coding RNAs identified in the *T. dendrolimi* genome, which covered 0.26 Mb and represented 0.12% of the genome (Table S6).

### 3.3. Orthology and Phylogenetic Analysis

**Figure 1:**
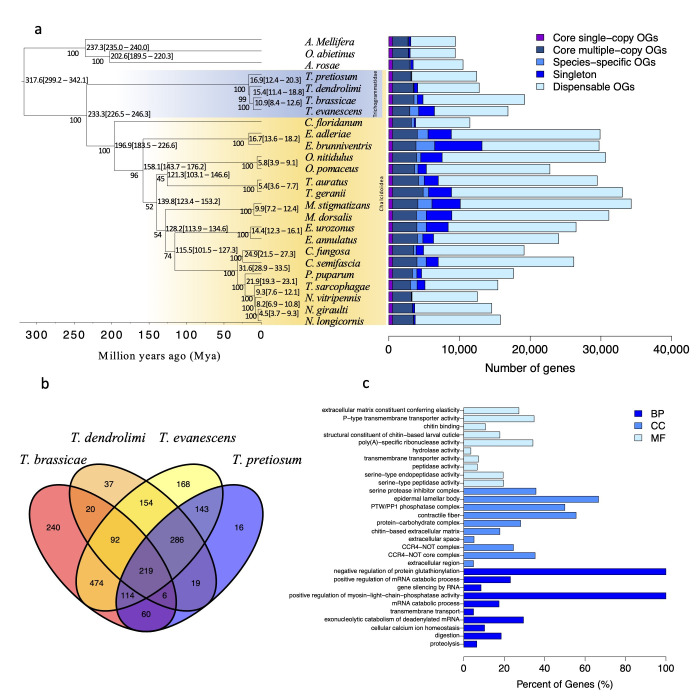
Comparative genomic analysis of the parasitoid *T. dendrolimi*. (**a**) A maximum likelihood phylogenetic tree (left panel) and gene ontology analysis (right panel). The phylogenetic tree was constructed for *T. dendrolimi*, 3 other *Trichogramma* species, 18 representative species of Chalcidoidea and 3 species from other hymenopteran groups *Apis mellifera*, *Orussus abietinus* and *Athalia rosae* as outgroups. The numbers on nodes indicate divergence times (Mya), with error bars showing 95% credit intervals. The numbers on branches indicate bootstrap support (1000 replicates). The length of branch indicates the divergence time. The bars on the right panel are showing the number of genes in the core orthogroups (OGs), dispensable OGs, species-specific OGs and singleton genes, as delineated by orthoFinder. (**b**) Venn diagram of specific OGs in *T. dendrolimi* and 3 other *Trichogramma* species. (**c**) GO enrichment classification of OGs shared by *T. dendrolimi* and 3 other *Trichogramma* species. The bars are showing the top 10 significant GO categories (*p* < 0.05) in biological process (BP), cellular component (CC) and molecular function (MF), respectively.

We used *T. dendrolimi*, 3 other *Trichogramma* species, 18 representative species of Chalcidoidea and 3 hymenopteran species, *Apis mellifera*, *Orussus abietinus* and *Athalia rosae,* as outgroups for phylogeny and orthology analysis. The phylogenetic tree was constructed based on 13,125 single-copy genes identified within *T. dendrolimi* and 24 other hymenopteran species (Figure 1a, left panel). The amino acid sequences of single-copy genes were aligned and concatenated for the following phylogenetic tree construction. Within Chalcidoidea, the four *Trichogramma* species were found in their own clade as the basal lineage, sister to all the other species of Chalcidoidea, which is consistent with the previous finding that the family Trichogrammatidae is one of the earliest branching families of Chalcidoidea [20]. In the other lineage of Chalcidoidea, *Copidosoma floridanum* was the basal lineage and Eurytomidae, Ormyridae, Torymidae, Megastigmidae, Eupelmus and Pteromalidae were found to subsequently diverge. Among the four *Trichogramma* species, *T. brassicae* and *T. evanescens* were closely clustered and *T. dendrolimi* and *T. pretiosum* were found to subsequently diverge. *T. brassicae* and *T. evanescens* diverged from *T. dendrolimi* approximately 15.4 million years ago and *T. dendrolimi* diverged from *T. pretiosum* about 16.9 million years ago.

Using the results of OrthoFinder, the number of genes in the core orthogroups (OGs) (including single-copy core OGs and variable-copy core OGs), dispensable OGs, species-specific OGs and singleton genes were estimated (Figure 1a, right panel). The core OGs, the dispensable OGs and species-specific OGs are the orthogroups present in all 25 species, absent in at least one species and only present in one species, respectively. Across the 25 species, there were 37,142 OGs identified in total. The average gene number per OGs was 12.7 genes. The largest OG had 1066 genes and the second largest OG had 883 genes. A total of 2438 OGs were present in all species used in our analysis, including 525 single-copy OGs. The number of genes in dispensable OGs and species-specific OGs were higher for species with more annotated protein-coding genes than the others. A total of 12,807 OGs was present in *T. dendrolimi* and three other *Trichogramma* species analyzed in this study, of which 2048 OGs were specific in four *Trichogramma* species, and GO enrichment analysis revealed that these *Trichogramma* specific genes were enriched in GO terms related to spectrin binding, localization, protein localization to plasma membrane, cytoskeleton organization and neuron projection (*p* < 0.05, Table S7). Further analysis was performed on these *Trichogramma* specific genes to identify *Trichogramma* core OGs and species-specific OGs. There were 219 OGs shared by *T. dendrolimi* and three other *Trichogramma* species (Figure 1b) and GO enrichment analysis revealed that core genes were enriched in digestion, proteolysis, transmembrane transport and structural constituent of chitin-based larval cuticle (*p* < 0.05, Figure 1c, Table S8). *T. dendrolimi* and *T. pretiosum* had less than 50 species-specific OGs, while *T. brassicae* and *T. evanescens* had more than 150 species-specific OGs (Figure 1b). In *T. dendrolimi*, 92 genes assigned to 37 OGs were enriched in asparagine biosynthetic process, regulation of intracellular pH, lactate transmembrane transport and response to cold (*p* < 0.05, Table S9).

### 3.4. Gene Family Expansions and Contractions

**Figure 2:**
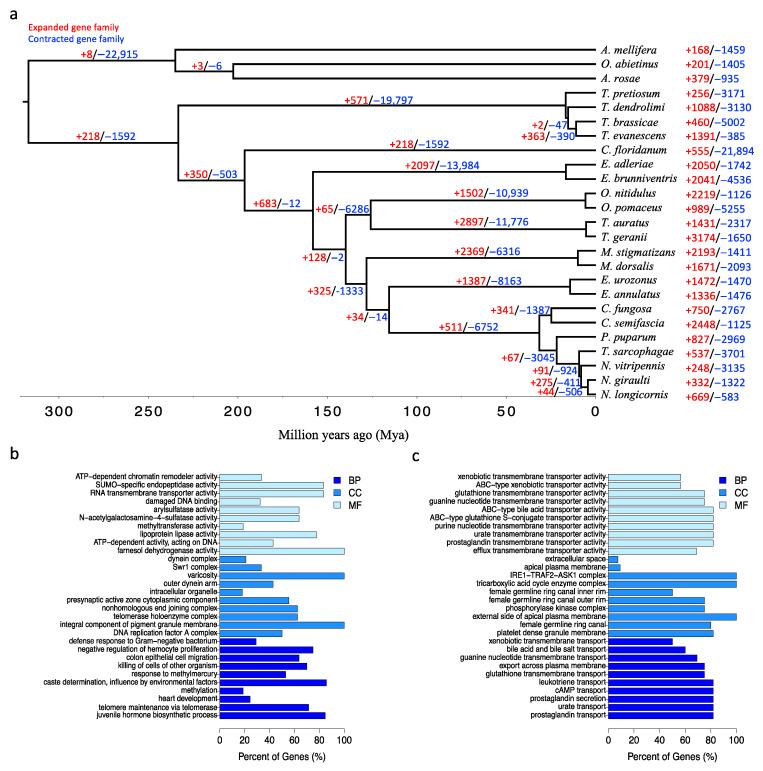
Analysis of gene family evolution analysis between genomes of *T. dendrolimi* and 24 other hymenopterans. (**a**) Numbers of gene families that have significantly expanded and contracted at branches across the phylogenetic tree. The number of significantly expanded and contracted gene families is indicated in red and blue, respectively. The length of branch indicated the divergence time. (**b**) GO enrichment classification of significantly expanded gene families of *T. dendrolimi*. (**c**) GO enrichment classification of significantly contracted gene families of *T. dendrolimi*. The bars are showing the top 10 significant GO categories (*p* < 0.05) in biological process (BP), cellular component (CC) and molecular function (MF), respectively.

Using CAFÉ, the gene family expansion and contraction in *T. dendrolimi* were estimated by comparison with those in 24 other hymenopteran species. The numbers of the expanded and contracted families were presented along the branches in the phylogeny (Figure 2a). A total of 1088 and 3130 gene families were expanded and contracted in *T. dendrolimi*, respectively. *T. pretiosum* and *T. brassicae* had lower numbers of significantly expanded gene families compared with *T. evanescens* and *T. dendrolimi* (256 families in *T. pretiosum*, 460 families in *T. brassicae* and 1391 families in *T. evanescens*). The significantly expanded gene families of *T. dendrolimi* were overrepresented for several GO terms related to development and regulatory process: juvenile hormone biosynthetic process, negative regulation of hemocyte proliferation, methylation and caste determination (Figure 2b, Table S10), while the significantly contracted gene families of *T. dendrolimi* showed GO enrichment in transport processes including prostaglandin transport, cAMP transport, leukotriene transport, glutathione transmembrane transport, export across plasma membrane, bile acid and bile salt transport and xenobiotic transmembrane transport (Figure 2c, Table S11).

### 3.5. Olfactory-Related Genes

**Figure 3:**
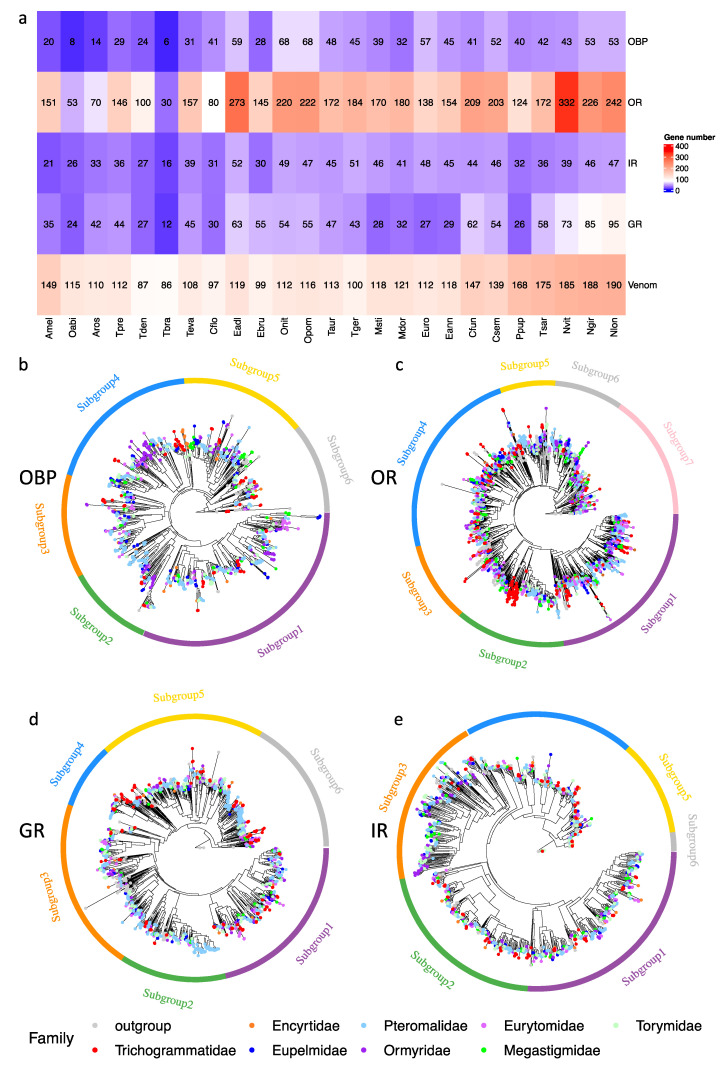
The comparison and analysis of the evolution of olfactory and venom genes in *T. dendrolimi* and 24 other hymenopterans. (**a**) The number of olfactory-related and venom genes. The description of species abbreviations has been provided in the method. (**b**) Maximum-likelihood phylogenetic tree of OBPs in *T. dendrolimi* and 24 other hymenopteran species. (**c**) Maximum-likelihood phylogenetic tree of ORs in *T. dendrolimi* and 24 other hymenopteran species. (**d**) Maximum-likelihood phylogenetic tree of GRs in *T. dendrolimi* and 24 other hymenopteran species. (**e**) Maximum-likelihood phylogenetic tree of IRs in *T. dendrolimi* and 24 other hymenopteran species. In the above phylogenetic trees, 25 hymenopteran species were reclassified as 9 groups. Different colors of tip nodes indicate different hymenopteran families. The tip nodes of Trichogrammatidae are highlighted in red. Amel, Outgroup; Oabi, Outgroup; Aros, Outgroup; Tpre, Trichogrammatidae; Tden, Trichogrammatidae; Tbra, Trichogrammatidae; Teva, Trichogrammatidae; Cflo, Encyrtidae; Eadl, Eurytomidae; Ebru, Eurytomidae; Onit, Ormyridae; OPom, Ormyridae; Taur, Torymidae; Tger, Torymidae; Msti, Megastigmidae; Mdor, Megastigmidae; Euro, Eupelmidae; Eann, Eupelmidae; Cfun, Pteromalidae; Csem, Pteromalidae; Ppup, Pteromalidae; Tsar, Pteromalidae; Nvit, Pteromalidae; Ngir, Pteromalidae; Nlon, Pteromalidae.

We identified OBP, OR, IR and GR profiling in *T. dendrolimi* and 24 other hymenopteran species, with uniform methods combining BLAST and HMM (Figure 3a). *T. dendrolimi* was found to harbor 24 OBPs, 100 ORs, 27 GRs and 27 IRs. Compared with *T. dendrolimi*, *T. pretiosum* and *T. evanescens* had more olfactory-related genes, while *T. brassicae* harbored a significantly reduced number of olfactory-related genes.

OBPs are small water-soluble chemoreceptor proteins that recognize, bind and transfer odor molecular pheromones to the transmembrane chemoreceptors (ORs, IRs and GRs) to activate olfactory receptor neurons and to stimulate behavioral responses. The four *Trichogramma* species contained 6–31 OBPs, in which *T. brassicae* harbored only 6 OBPs, the smallest number of OBPs among all investigated hymenopteran species, while the other three *Trichogramma* species had approximately 30 OBPs. The *Ormyrus* species (*Ormyrus nitidulus* and *Ormyrus pomaceus*) had the largest the number of OBPs (68 OBPs) and other Chalcidoidea species had 28–59 OBPs (Figure 3a). Phylogenetic analysis clustered the OBPs genes of all investigated species into six subgroups (Figure 3b). The OBPs of *Trichogramma* species were distributed in all six subgroups but were primarily found in their own species-specific lineages. Such species-specific lineages were also found in other investigated species, thereby revealing several gene duplication events.

ORs and GRs are G-protein-coupled receptors, containing seven transmembrane domains and are responsible for recognition and discrimination of odorant signals [63,64]. The ORs in all 25-investigated hymenopteran species exhibited dramatic dynamics in the gene family size. The *Nasonia*, *Ormyrus* and *Cecidostiba* species were found to contain more than 200 ORs and the other Chalcidoidea species had 100–200 ORs, except *T. brassicae,* which only harbored 30 ORs (Figure 3a). The ORs were classified into seven subgroups (Figure 3c). The ORs of the *Trichogramma* species, were unevenly distributed in this phylogeny tree. A major species-specific lineage was found in subgroup 2, indicating these ORs might have undergone an extensive lineage-specific gene duplication. The GRs of all the investigated hymenopteran species also showed variations in the gene family size. The *Trichogramma* species, except *T. brassicae* (only 12 GRs), contained 27–45 GRs. Other Chalcidoidea species had 27–95 GRs, in which the *Nasonia*, Ormyrus, *Cecidostiba* and *Eurytoma* species were found to contain more than 50 GRs (Figure 3a). The phylogenetic analysis of GRs classified these genes into six subgroups (Figure 3d). Unlike the ORs, the GRs of the *Trichogramma* species were generally evenly distributed in the six subgroups.

IRs are members of the ionotropic glutamate receptor (IgluR) family, which have been identified to be involved in not only olfactory functions but also taste, temperature and humidity perception [65]. Most of the investigated hymenopteran species had about 20–50 IRs, except *T. brassicae* (only 16 IRs) (Figure 3a). The IRs of the 25-investigated hymenopteran species were clustered into six subgroups (Figure 3e). Interestingly, IRs of the *Trichogramma* species were widely distributed in the phylogenetic tree, except the subgroup 3, which only contained 7 IRs.

### 3.6. Venom-Related Genes

Venoms are important effectors injected by female parasitoids during oviposition to regulate host immunity response and development to promote the growth, development and survival of their offspring [66,67]. We manually annotated venom protein genes of *T. dendrolimi* and 24 other hymenopteran species through BLAST searches against publicly available venom proteins sequences of *Apis mellifera* (Lepidoptera: Apidae) [68] and several parasitoid wasps, including *Pteromalus puparum* (Hymenoptera: Pteromalidae) [69], *Nasonia vitripennis* (Hymenoptera: Pteromalidae) [70], *Chelonus inanitus* (Hymenoptera: Braconidae) [71], *Leptopilina boulardi* and *Leptopilina heterotoma* (Hymenoptera: Figitidae) [72] using a stringent cutoff. Using this uniform blast method, we identified a varied number of venom genes in these 25 hymenopteran species (Figure 3a). The four *Trichogramma* species contained the minimum number of venom genes (86–112 venom genes), while seven species of Pteromalidae and *Apis mellifera* had the maximum number of venom genes (139–190 venom genes). The rest of the hymenopteran species had about 120 venom genes. Further analysis revealed that majority of the variations in venom gene numbers were from the hits matched against venom gene sequences from *Apis mellifera*, *Nasonia vitripennis* and *Pteromalus puparum* (Table S12), indicating most of the venom genes from these three species might be species- or lineage-specific venom genes. However, the number of hits matched against the *Leptopilina boulardi* and *Leptopilina heterotoma* was about 40–50 for the 25 hymenopteran species, revealing that the two *Leptopilina* species contained more shared venom genes rather than species-specific venom genes. Further analysis of the venom genes from the four *Trichogramma* species showed that about 90% of hits matched against *Leptopilina boulardi* and *Leptopilina heterotoma;* the detailed description of these venom genes have been provided in Table S13. Then, we performed GO enrichment analysis on the venom genes of *T. dendrolimi* and found that they were enriched in antioxidant activity, tricarboxylic acid cycle, response to oxidative stress and cell redox homeostasis (*p* < 0.05, Table S14).

## 4. Discussion

Egg parasitoids in the genus *Trichogramma* are one of the most used biocontrol agents worldwide through inundative releases [73]. Due to intensive research on and effective industrialization of *Trichogramma* in the last decades, China is leading the world in *Trichogramma*-based biological control programs such as diapause manipulation, optimal use of factitious hosts and new application methods [9]. Among the commonly used *Trichogramma* species, *T. dendrolimi* is one of the most successfully industrialized *Trichogramma* species used to control agricultural and forestry pests because of its broad distribution, the wide host range and optimized mass-rearing method on eggs of the Chinese oak silkworm, *A. pernyi* [4,9]. So far, a number of studies have provided insight into the biology and ecology of *T. dendrolimi,* leading to successful biological control in the field [4,6,9,74]. However, the genome information of this important *Trichogramma* species is lacking and restricts further exploration of the molecular mechanism underlying its host recognition and parasitism of *T. dendrolimi*.

To date, the whole genomes have been obtained for many parasitoid wasp species [19], most of which were parasitoid wasps developing in larval or pupal hosts, while very few egg parasitoid wasps have been sequenced and assembled for the whole genome sequences. The limited genome information on egg parasitoid wasps has hindered the unraveling of the molecular mechanisms underlying their parasitism. For example, most studies conducted on parasitoids in the genus *Trichogramma*, have focused on the identification of traits that contribute to successful biological control, while the underlying molecular mechanisms remain poorly understood, which is partially due to the lack of a comprehensive genome data for this species. So far, three *Trichogramma* genomes (*T. pretiosum*, *T. brassicae* and *T. evanescens*) are publicly available on InsectBase 2.0 [51] and NCBI, which were sequenced mainly on Illumina platforms. Compared with these three *Trichogramma* genomes, our genome was sequenced using a combination of Illumina and PacBio platforms and the final genome had higher continuity and completeness. Notably, the three *Trichogramma* species with publicly available genomes are not commonly used in China. Therefore, our genome will serve as the first whole genome sequence of the *Trichogramma* species in China and provide comprehensive genomic information to identify the species-specific features.

*T. dendrolimi* and the three other *Trichogramma* species have moderate-size genomes (213.67–235.41 Mb) among the hymenopterans with sequenced genomes (180–340 Mb) [19,75,76]. The content of repetitive sequences (~30%) of the *Trichogramma* species (*T. dendrolimi* and *T. pretiosum*) is nearly the same as that of most chalcidoids. *Copidosoma floridanum* contains about 31.12% repetitive sequences in its genome (data from NCBI *Copidosoma floridanum* Annotation Release 101) and *Nasonia* species were also found to harbor about 30% of repetitive sequences in their genomes [18]. We predicted 12,785 genes in the genome of *T. dendrolimi*, which is within the range of 12,000–20,000 genes reported for hymenoptera genomes [19]. Hymenoptera genomes are known for their low GC content, ranging from 30–45% [77], and that of chalcidoids genomes had even lower GC content, ranging from 20–30%. Such low GC content is a common feature of hymenoptera genomes and probably due to GC-biased gene transformation and a high recombination rate [78].

Our phylogeny analyses found that the family Trichogrammatidae was clustered in its own clade as the basal lineage, sister to all the other species of Chalcidoidea, which agrees well with previous finding that this family Trichogrammatidae is one of the earliest branching families of Chalcidoidea [20]. A previous phylogeny study based on transcriptomes found that Mymaridae and Trichogrammatidae, two small egg parasitoids, were placed as sister groups of all remaining Chalcidoidea, indicating that small body size and egg parasitoidism were possible ancestral traits of this super family [79]. Compared with the previous estimation by Ma et al. [80], the range of the estimated age of the divergence between *T. pretiosum* and three other *Trichogramma* species increased from 6.4 Mya to 16.9 Mya.

The number of olfactory-related genes showed dramatic variations among the studied hymenopteran species, supporting the differences among their chemosensory systems and biology. The four *Trichogramma* species harbored significantly different numbers of olfactory-related genes and the gene number was correlated with the host range of these *Trichogramma* species [81], indicating that the host-parasitoid interactions may affect the dynamics by the process of coevolution. To be noticed, the accurate identification of olfactory-related genes was determined by the available data and methods. The previous studies based on the whole genome sequence and transcriptome of *T. pretiosum* identified 22 OBPs and 105 ORs, while we found 29 OBPs and 146 ORs in *T. pretiosum,* using methods combining BLAST and HMM profiling. Different versions of the whole genome sequences and methods might result in different gene numbers.

OBPs are some of the most abundant proteins in insect olfactory organs, where they are involved in the first step of odorant detection and carrying odorant molecules to the olfactory receptors. Subsequently, the three transmembrane transporters, ORs, GRs and IRs, function as olfactory receptors to receive and discriminate these signals. We found an obvious contraction of OBPs, ORs, GRs and IRs in Trichogrammatidae compared with other species of Chalcidoidea, which may result from their very tiny body size and spending most of their lifetime inside the host eggs. The Pteromalidae family was found to harbor a high number of OBPs and olfactory receptors relative to other species of Chalcidoidea, which could be related to the need of this family to detect and discriminate between a number of diverse odors for food and reproduction or to avoid harmful substances [82]. The amino acid sequences of OBPs and ORs between *T. pretiosum* and *N. vitripennis* were reported to show high similarities [83], indicating that these conserved proteins may have similar functions in these two generalist parasitoid wasps, while the dramatically different number of these proteins between the two parasitoid wasps reflected considerable gene subfamily expansion in the *Nasonia* lineage [82].

The roles of the venom in parasitoids have evolved in parallel with their parasitic lifestyle. In ectoparasitoids, the venom often induce a long-term paralysis of the hosts [84], while endoparasitoid venoms are mainly involved in regulating the host physiology by suppressing immune responses or delaying or arresting host development [85]. So far, the functions of endoparasitoid venoms have been intensively studied in the larval or pupal parasitoids, while they are poorly understood in egg parasitoids. Egg parasitoids are idiobionts, which kill the host during oviposition [86]. Our analyses identified several venom genes showing an enrichment in terms related to antioxidant activity, response to oxidative stress and cell redox homeostasis, supporting the hypothesis that egg parasitoids inject oxidoreductase as venoms into the host to interfere with the host melanization process and production of cytotoxic compounds [87,88]. Although we identified venom genes in *T. dendrolimi* and 24 other hymenopteran species through a blast method using very stringent criteria, the further analysis showed that such a blast method was very biased because it could only identify venom genes that had homologs in databases with predicated functions. Nowadays, an increasing number of studies reported that there exist variations in venom compositions between different parasitoid species, even closely related species [24,72,73]. Therefore, a combination of transcriptomics and proteomics of venom glands are necessary to comprehensively understand the proteins in the venom that *Trichogramma* species inject into the host.

## 5. Conclusions

In this study, we provide a high-quality genome assembly of *T. dendrolimi*, an important biocontrol agent to control lepidopteran pests, through a combination method of Illumina short-read sequencing and single-molecule real-time (SMRT) long-read sequencing technologies. We analyzed the genetic relationships among *T. dendrolimi*, 3 *Trichogramma* species, 18 representative Chalcidoidea species and 3 outgroup hymenopteran species with publicly available genomes. We also investigated the expanded and contracted gene families in *T. dendrolimi* and found that a number of contracted gene families were involved in transport processes. In addition, we identified olfactory and venom-related genes in *T. dendrolimi* and 24 other hymenopteran species through a uniform blast method. Our study provides an important resource for future studies in comparative genomics, functional studies, parasitoid-host interaction and biological pest control.

## Figures and Tables

**Table 1:** The genomic feature of the assembly of *Trichogramma dendrolimi*.

Genomic Feature	*Trichogramma dendrolimi*
Length of the assembly (bp)	215,209,100
Maximum scaffold length (bp)	9,241,039
Number of scaffolds	316
Scaffold N50 (bp)	1,412,680
Number of genes	12,785
Average gene length (bp)	6407.6
Average coding sequence length (bp)	1389.1
Average exon length (bp)	268.5
Average intron length (bp)	1202.3
Average exon number	5.17
Total size of transposable elements (bp)	63,402,215
GC content	39.8%
BUSCO scores	C: 93.4% [S: 88.6%, D: 4.8%], F: 1.0%, M: 5.6%, *n*: 1367

The BUSCO scores indicated that 93.4%, 1.0% and 5.6% of the 1367 BUSCO genes within the insecta_odb10 BUSCO set were present in the assembled *T. dendrolimi* genome as complete (C), fragmented (F) and missing genes (M). Out of the 93.4% complete genes, 88.6% are single-copy genes (S) and 4.8% are duplicated genes (D).

## Data Availability

Data supporting the findings of this work are available within the paper and its Supplementary Materials. A reporting summary for this article is available as a Supplementary Materials. All other raw data are available from the corresponding author upon request. The genome assembly has been deposited in NCBI database under BioProject accession PRJNA900302, BioSample accession SAMN31686375 and GeneBank accession JAPMIB000000000. The raw data have been deposited in NCBI Sequence Read Archive under accession SRR22366802, SRR22366803, SRR22366804, SRR22366805, SRR22366806, SRR22366807, SRR22366808, SRR22366809 and SRR22366810. The nucleotide and amino acid sequences of protein coding genes and the gff file are publicly available at Cyverse (https://data.cyverse.org/dav-anon/iplant/home/moontree1985/analyses/Tden/Tden.cds; https://data.cyverse.org/dav-anon/iplant/home/moontree1985/analyses/Tden/Tden.pep; https://data.cyverse.org/dav-anon/iplant/home/moontree1985/analyses/Tden/Tden.gff, accessed on 1 January 2023).

## Supplementary Materials

The following supporting information can be downloaded at: https://www.mdpi.com/article/10.3390/insects14020144/s1, Figure S1: Comparison of genome size, GC content and gene number between *T. dendrolimi* and 24 other hymenopterans. Table S1: Comparison of assembly statistics between *T. dendrolimi* and three publicly available *Trichogramma* species; Table S2: Summary of library construction and sequencing output; Table S3: Comparison of gene models between *T. dendrolimi* and *T. pretiosum*; Table S4: Summary statistics of gene annotation; Table S5: Summary of TE superfamilies in *T. dendrolimi*; Table S6: The summary of non-coding RNAs in *T. dendrolimi* genome; Table S7: GO enrichment analysis of specific 2048 OGs in four *Trichogramma* species; Table S8: GO enrichment analysis of 219 OGs shared by *T. dendrolimi* and three other *Trichogramma* species; Table S9: GO enrichment analysis of 37 specific OGs in *T. dendrolimi*; Table S10: GO enrichment analysis of significantly expanded gene families in *T. dendrolimi*; Table S11: GO enrichment analysis of significantly contracted gene families in *T. dendrolimi*; Table S12; Summary of venom genes identified by BLASTP against the venom database from different species; Table S13: Summary of venom protein sequences of *T. dendrolimi* and three other *Trichogramma* species identified by BLASTP against the venom database; Table S14: GO enrichment analysis on specific venom genes of *T. dendrolimi*.
