# Supplementary material for: Genome Assembly and Comparative Analysis of the Egg Parasitoid Wasp Trichogramma dendrolimi Shed Light on the Composition and Evolution of Olfactory Receptors and Venoms
Source: Insects. 2023 Jan 31;14(2):144. doi: 10.3390/insects14020144 (PMC9960361; doi:10.3390/insects14020144)
Supplement: Supplementary file 1 [file insects-14-00144-s001.zip › Supplementary Files/Supplementary Figure S1 and Tables S1-S6.docx]

Supplementary Materials Figure S1 and Tables S1–S6

Genome Assembly and Comparative Analysis of the Egg
Parasitoid Wasp *Trichogramma dendrolimi* Shed Light
on the Composition and Evolution of Olfactory
Receptors and Venoms

Xue Zhang ^1^, Zhuo Jiang ^1^, Xilin Jiao ^1^, Yang Yu ^1^, Zhenan Wang ^2^, Yangyang Hou ^3^, Guohua Duan ^2^, Wenmei Du ^1^, Changchun Ruan ^1^, Junjie Zhang ^1,^* and Ying Hu ^1,^*

^1^ Engineering Research Center of Natural Enemies, Institute of Biological Control,
Jilin Agricultural University, Changchun 130118, China; zhangxue871013@163.com (X.Z.);
jzjz8968@163.com (Z.J.); j18943100339@163.com (X.J.); m15684130039@163.com (Y.Y.);
wenmeid@jlau.edu.cn (W.D.); ruanchangchun@126.com (C.R.)

^2^ Department of Plant Pathology, College of Plant Protection, Jilin Agricultural University,
Changchun 130118, China; wznemail@163.com (Z.W.); ghduan1990@163.com (G.D.)

^3^ Department of Entomology, College of Plant Protection, Jilin Agricultural University,
Changchun 130118, China; tiantian1214418@163.com

* Correspondence: junjiezh@126.com (J.Z.); huying@jlau.edu.cn (Y.H.); Tel.: +86-0431-8453-2834 (Y.H.)

**Figure S1.** Comparison of genome size, GC content and gene number between *T. dendrolimi* and 24 other hymenopterans.

**Table S1.** Comparison of assembly statistics between *T. dendrolimi* and three publicly available *Trichogramma* species.

| **Assembly features** | ***T. dendrolimi*** | ***T. pretiosum*** | ***T. brassicae*** | ***T. evanescens*** |
| --- | --- | --- | --- | --- |
| **Sequencing technology** | PacBio+Illumina | Illumina | PacBio+Illumina | Illumina |
| **Genome coverage** | 271.87 x | 232.7 x | 162 x | 100 x |
| **Assembly level** | Scaffolds | Scaffolds | Contigs | Scaffolds |
| **Genome size** | 215.21 Mb | 195.09 Mb | 235.41 Mb | 213.67 Mb |
| **Number of contigs** | 451 | 7879 | 1570 | 149,888 |
| **Number of scaffolds** | 316 | 357 | NA | 146,286 |
| **Scaffold N50** | 1.41 Mb | 3.71 Mb | NA | 0.38 Mb |
| **Contig N50 (bp)** | 1.13 Mb | 0.79Mb | 0.56 Mb | 0.27 Mb |
| **BUSCO score** | C: 93.4% | C: 91.8% | C: 95.9% | C: 96.8% |
| **GC content** | 39.8% | 39.9% | 39.8% | 39.8% |
| **Repeat content** | 29.5% | 30.3% | NA | NA |
| **Number of protein coding genes** | 12,785 | 12,928 | 16,905 | NA |

**Table S2.** Summary of library construction and sequencing output.

| **Pair-End Libraries** | **Insert Size** | **Total Data (G)** | **Sequence Coverage (x)** | **Read Length (bp)** |
| --- | --- | --- | --- | --- |
| **Illumina reads** | 250 bp | 6.82 | 30.46 | 150 |
|  | 450 bp | 9.50 | 42.43 |  |
|  | 2 kb | 11.87 | 52.60 |  |
|  | 5 kb | 9.51 | 42.49 |  |
|  | 10 kb | 6.41 | 28.64 |  |
| **PacBio reads** | 20 kb | 16.86 | 75.26 | - |
| **Total** | - | 60.9 | 271.87 | - |

**Table S3.** Comparison of gene models between *T. dendrolimi* and *T. pretiosum.*

|  | ***T. dendrolimi*** | ***T. pretiosum*** |
| --- | --- | --- |
| Number of gene models | 12785 | 12928 |
| Average gene length (bp) | 6407.61 | 7253.97 |
| Average CDS length (bp) | 1389.05 | 1681.03 |
| Average exons per gene | 5.17 | 5.80 |
| Average exon length (bp) | 268.47 | 289.75 |
| Average intron length (bp) | 1202.33 | 1160.61 |

**Table S4.** Summary statistics of gene annotation.

| **Database** | | **Annotated Number** | **Annotated Percent (%)** |
| --- | --- | --- | --- |
| **NR** | | 11566 | 90.5 |
| **Swiss-Prot** | | 9480 | 74.1 |
| **KEGG** | | 9210 | 72.0 |
| **InterPro** | **All** | 9851 | 77.1 |
|  | **Pfam** | 9004 | 70.4 |
|  | **GO** | 7259 | 56.8 |
| **Annotated** | | 11657 | 91.2 |
| **Total** | | 12785 | - |

**Table S5.** Summary of TE superfamilies in *T. dendrolimi.*

|  | **Denovo + Repbase** | | **TE proteins** | | **Combined TEs** | |
| --- | --- | --- | --- | --- | --- | --- |
|  | **Length (bp)** | **Percentage of genome** | **Length (bp)** | **Percentage of genome** | **Length (bp)** | **Percentage of genome** |
| **DNA transposon** | 11286931 | 5.24% | 4376023 | 2.03% | 13809113 | 6.41% |
| **Retrotransposon** | 35623383 | 16.55% | 13962148 | 6.48% | 37939191 | 17.63% |
| LTR | 22877340 | 10.63% | 7539641 | 3.50% | 23600717 | 10.97% |
| LINE | 12503971 | 5.81% | 6422507 | 2.98% | 14096402 | 6.55% |
| SINE | 242072 | 0.11% | 0 | 0 | 242072 | 0.11% |
| **Other** | 773 | 0.00% | 0 | 0 | 773 | 0.00% |
| **Unknown** | 17457139 | 8.11% | 0 | 0 | 17457139 | 8.11% |
| **Total** | 60057914 | 27.91% | 18272463 | 8.49% | 63402215 | 29.46% |

**Table S6.** The summary of non-coding RNAs in *T. dendrolimi* genome.

| **Type** | | **Copy** | **Average length (bp)** | **Total length (bp)** | **% of genome** |
| --- | --- | --- | --- | --- | --- |
| **miRNA** | | 447 | 164.15 | 73377 | 0.034 |
| **tRNA** | | 305 | 75.72 | 23095 | 0.011 |
| **rRNA** | **rRNA** | 279 | 267.20 | 74549 | 0.034 |
|  | **18S** | 174 | 368.04 | 64039 | 0.029 |
|  | **28S** | 24 | 143 | 3432 | 0.002 |
|  | **5.8S** | 25 | 99.08 | 2477 | 0.001 |
|  | **5S** | 56 | 82.16 | 4601 | 0.002 |
| **snRNA** | **snRNA** | 110 | 127.56 | 14032 | 0.006 |
|  | **CD-box** | 17 | 171 | 2907 | 0.001 |
|  | **HACA-box** | 9 | 139.55 | 1256 | 0.001 |
